# Cervical Cancer Brachytherapy Dose Escalation Protocol: Analysis of Early Data Treatments According to EMBRACE II Protocol

**DOI:** 10.7759/cureus.107803

**Published:** 2026-04-27

**Authors:** Michail Gouliaris, Argyro Papalaskari, Pantelis Katsapanteras, Lydia Zoglopitou, Konstantina Mponiou, Anna Makridou, Martha Charalampidou

**Affiliations:** 1 Medical Physics Department, Cancer Hospital of Thessaloniki “Theagenio”, Thessaloniki, GRC; 2 Radiation Oncology Department, Cancer Hospital of Thessaloniki “Theagenio”, Thessaloniki, GRC

**Keywords:** cervical cancer, dosimetric analysis, embrace ii protocol, high-dose rate (hdr) brachytherapy, image guided radiotherapy

## Abstract

Background: Cervical cancer is one of the most prevalent malignancies among women, particularly in developing countries. The combination of external beam radiotherapy (EBRT) and brachytherapy (BT) is the preferred treatment modality for patients with advanced-stage disease. High-dose rate brachytherapy (HDR BT) plays a crucial role in the overall treatment by delivering high radiation doses to the target volume while minimizing exposure to surrounding healthy tissues.

Purpose: The statistical analysis focuses on the correlation of dosimetric parameters with dose constraints and the incremental dose increase per BT session. Additionally, it compares the total dose received by each patient from EBRT and BT in the high-risk clinical target volume (HR-CTV) and organs at risk (OARs) with the dose constraints of the modern EMBRACE II (European study on MRI-guided BRAchytherapy in locally advanced CErvical cancer) protocol. This study aims to highlight the significance of BT in cervical cancer treatment and provide insights to enhance treatment efficacy.

Materials and methods: This study retrospectively analyzed the treatment data of 34 patients with locally advanced cervical cancer. Each patient initially received EBRT, followed by three HDR BT sessions, with the dose per session initially set at 7 Gy and later increased to 8 Gy. Data for this study were collected using a treatment planning system (TPS) software. For each patient, the dose received by 90% of the HR-CTV (D90) and the dose received by the 2 cc volume of each OAR (D2cc) were recorded and converted to an equivalent dose in 2 Gy fractions (EQD2) using α/β=10 Gy for the tumor and α/β=3 for each OAR.

Results: The mean D90 for HR-CTV increased significantly with the transition from 7 Gy to 8 Gy per fraction, resulting in improved target coverage. A total of seven out of 34 treatment plans achieved the recommended-acceptable total dose limit (EBRT + BT) ≥ 85 Gy EQD2 for HR-CTV, and 13 out of 34 treatment plans achieved a total dose between 80 and 85 Gy. For all 34 treatment plans, the OARs complied with the EMBRACE II dose constraints: D2cc < 90 Gy for the bladder, < 75 Gy for the rectum, and < 70 Gy for the sigmoid.

Conclusion: The dosimetric results indicate that CT-based BT (IBBT) planning can achieve adequate tumor coverage while maintaining OAR doses within the acceptable limits of the EMBRACE II protocol. These findings support the ongoing optimization of individualized treatment planning and highlight the importance of having commonly defined limits that are regularly updated.

## Introduction

Among the most prevalent malignancies in the pelvic region is cervical cancer, predominantly affecting middle-aged women. Accurate diagnosis and advanced treatment modalities can facilitate its management. For patients with cervical cancer beyond stage II, treatment typically begins with external irradiation of the tumor, followed by brachytherapy (BT) sessions [1]. A significant advantage of BT over external beam radiotherapy (EBRT) is that, in BT, the radioactive source is placed inside the patient, allowing for high doses of radiation to be delivered locally. This approach effectively eradicates cancer cells while minimizing damage to surrounding healthy tissues and organs [2,3]. For a BT session to be deemed successful, dosimetric analysis is essential to ensure the radiation dose is delivered safely and effectively [4]. This BT procedure can prevent potential tissue damage and reduce treatment-related complications. This approach aims to optimize dose distribution and minimize exposure to surrounding organs at risk (OARs). Recent studies, such as the EMBRACE II protocol, have significantly contributed to optimising the dosimetric approach to BT, thereby improving treatment accuracy and patient outcomes [5].

Recent clinical evidence suggests that 7 Gy per BT session may be insufficient for achieving the recommended cumulative equivalent dose in 2 Gy fractions (EQD2) ≥ 85 Gy for high-risk clinical target volume (HR-CTV), particularly in patients with larger tumor burdens or suboptimal response after EBRT. Several institutions have reported improved target coverage using 8 Gy fractions without exceeding OAR dose constraints. Therefore, evaluating the clinical feasibility and dosimetric impact of increasing the BT dose from 7 Gy to 8 Gy is warranted. This study aligns with the dosimetric principles and dose thresholds introduced by the EMBRACE II protocol, enabling direct comparison between clinical practice and contemporary international standards.

The primary objective of this study was to evaluate the feasibility and dosimetric impact of increasing the HDR-BT dose per session from 7 Gy to 8 Gy in cervical cancer treatment. Furthermore, the secondary objectives were to assess the cumulative EQD2 dose delivered to the HR-CTV and OARs (EBRT+BT) and compare these with the EMBRACE II dose constraints, to examine how dose escalation affects D90 (HR-CTV) and D2cc of major OARs, and to explore whether a higher dose per BT session improves tumor coverage without exceeding OAR toxicity thresholds.

## Materials and methods

Patient selection

This study included 34 female patients over the age of 18 who had been diagnosed with biopsy-confirmed stage II-IV cervical cancer, as classified by the International Federation of Gynecology and Obstetrics (FIGO) [6].

Brief report of the overall treatment

The initial treatment protocol for cervical cancer involves EBRT over a designated timeframe [7], when patients receive a prescribed dose of 45-50 Gy in 25 fractions (1.8-2.0 Gy per fraction) followed by three sessions of high-dose rate brachytherapy (HDR-BT), one session conducted per week. For the cumulative dose calculation, the EBRT contribution to the HR-CTV and OARs was individually converted to its EQD2 for each patient, ensuring consistent integration with the BT component as per EMBRACE II guidelines. For a standard 45 Gy schedule (1.8 Gy/fraction), the EBRT contribution was calculated at 44.3 Gy for the HR-CTV (α/β=10 Gy) and 43.2 Gy for OARs (α/β=3 Gy). For schedules utilizing 50 Gy (2 Gy/fraction), the physical dose was equal to the EQD2 (50 Gy) for both structures. This individualized approach accounts for the radiobiological differences between the treatment schedules.

Clinical assessment and preplanning of intrauterine cervical brachytherapy

Conducting a clinical assessment and pre-planning for endometrial BT by the Oncologist and the Medical Physicist is crucial for optimal therapy efficacy. This process involves reviewing each patient’s MR images obtained before initial EBRT and after the completion of EBRT [8], emphasizing tumor reduction and evaluating infiltration into surrounding tissues. The dimensions of the cervical canal and endometrial cavity are measured to ensure the selection of the appropriate intrauterine catheter length and angle, as well as the ovoids’ diameter. All procedures were performed using the Geneva™-Universal Gynecological Applicator (Elekta AB, Stockholm, Sweden). The D2cc from EBRT for each OAR (e.g., bladder, rectum, sigmoid) is determined in order to establish patient-specific dose constraints for each BT session.

EMBRACE II

The EMBRACE II (European study on MRI-guided BRAchytherapy in locally advanced CErvical cancer) research project primarily aims to evaluate and enhance treatment strategies combining EBRT and BT for cervical cancer [5]. This study, authored and published in 2018 by a consortium of European and International scientists, was conducted under the auspices of GEC-ESTRO (Gynaecological-European Society for Radiotherapy and Oncology). The study presents treatment methodologies integrating EBRT with MRI-guided BT. The protocol allows the use of a computed tomography (CT) scanner for BT sessions when an MRI is unavailable. The protocol establishes guidelines for dose to the HR-CTV and OARs, specifying doses to ensure treatment efficacy while minimizing side effects. The proposed dose limits for each structure encompass the total dose, which includes the combined dose from both EBRT and BT sessions. This can be accomplished by converting the dose per BT session into an EQD2, establishing a standardized comparison across treatment phases. According to the EQD2 formula:

$\mathrm{EQD}_{2} = D \cdot \left( \frac{d + \alpha/\beta}{2 + \alpha/\beta} \right)$

Where: D = total dose (D = nd), n = number of fractions, d = dose per fraction (Gy), and α/β = tissue-specific parameter (Gy).

In cervical cancer treatment, EQD2 and biologically effective dose (BED) calculations help in combining EBRT and brachytherapy doses, as they provide a biologically consistent comparison of treatments delivered with different fractionation schemes. They are especially important in adhering to international treatment guidelines such as EMBRACE II [5]. Typical values are α/β = 10 Gy for tumors and α/β = 3 Gy for OARs, as commonly reported in radiobiology literature [9].

The thresholds for the target tumor and the surrounding cervical structures are [10] for the HR-CTV, (i) acceptable therapeutic dose limit: total dose (EBRT+BT) > 85 Gy EQD2, and (ii) ideal therapeutic dose limit: total dose (EBRT+BT) around 90-95 Gy EQD2.

The cumulative dose limit for the target tumor is determined by combining doses from EBRT and all BT sessions. EBRT delivers 45-50 Gy, while each BT session evaluates the D90 parameters, converted to an equivalent EQD2 dose. The sum of these EQD2 doses and EBRT dose constitutes the total administered dose to the HR-CTV: (i) For the bladder, ideal dose limit: D2cc ≤ 80 Gy EQD2 and acceptable dose limit: D2cc ≤ 90 Gy EQD_2_; (ii) for the rectum, ideal dose limit: D2cc ≤ 65 Gy EQD2 and acceptable dose limit: D2cc ≤ 75 Gy EQD2; and (iii) for the sigmoid, ideal dose limit D2cc ≤ 70 Gy EQD2 and acceptable dose limit: D2cc ≤ 75 Gy EQD2.

Data extraction

Following applicator placement, a CT scan is conducted to acquire the requisite images for planning. The HR-CTV and OARs are delineated using the TPS (Oncentra Brachy, version 4.6.3.12, Elekta AB, Stockholm, Sweden) [11,12]. Axial, sagittal, and coronal CT images are used to enhance the Radiation Oncologist’s (RO) contouring accuracy. Plan optimization is performed using graphical optimization, with manual dwell-time adjustments. The Medical Physicist selects the positions and dwell times of the Ir-192 source during treatment planning. All generated treatment plans are subsequently reviewed and approved by the RO as clinically acceptable. Dose Volume Histogram (DVH) calculation provides the dosimetric data [13], including the D90 and D2cc parameters.

Statistical analysis

Dosimetric data from the cohort of 34 patients were systematically collected and organized in a Microsoft Excel (Microsoft Corporation, Redmond, WA, USA) spreadsheet. The dosimetric data analysis included a dose escalation study of the BT plans for the 34 patients, over a 47-month period, followed by the evaluation of the cumulative (EBRT+BT) dose administered to each patient’s HR-CTV. Further statistical analysis was performed using IBM SPSS Statistics for Windows, version 29.0 (IBM Corp., Armonk, NY, USA). Normality of the data distribution was assessed using the Shapiro-Wilk test, given the relatively small sample size. Since the data followed a normal distribution, differences between the two independent groups (7 Gy and 8 Gy) were evaluated using an independent samples t-test. Homogeneity of variances was assessed using Levene's test. Results are presented as mean dose ± standard deviation (SD) along with 95% confidence intervals (CI). To quantify the magnitude of the difference, Cohen's d effect size was also calculated. A p-value < 0.05 was considered statistically significant.

## Results

Dose escalation

**Figure 1:**
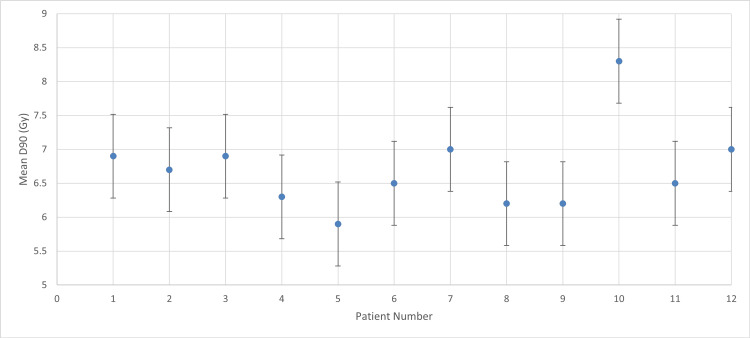
Mean D90 with 7 Gy prescribed dose D90: dose received by 90% of the high-risk clinical target volume

The RT department policy is to administer three BT sessions over three weeks, one session per week. EBRT delivered a prescribed physical dose of 45-50 Gy in 25 fractions (1.8-2.0 Gy per fraction) to the pelvic target volume, including the uterus and cervical region. In selected cases, a boost of 50 Gy was delivered to involved lymph nodes. To ensure dosimetric accuracy, the EBRT contribution was individually converted to EQD2 for each patient. Specifically, for the 45 Gy schedule (1.8 Gy/fraction), the HR-CTV received an EQD2 contribution of approximately 44.3 Gy (α/β = 10) prior to BT, while the OARs received approximately 43.2 Gy EQD2 (α/β = 3). In cases where 50 Gy (2.0 Gy/fraction) was administered, the EQD2 contribution was 50 Gy for both the target and the OARs, as the dose per fraction equals the reference dose of 2 Gy. In the subsequent BT phase, the first 12 patients received 7 Gy per BT session, and the remaining 22 patients received 8 Gy per BT session. Initially, the prescribed dose per BT session was set at 7 Gy, based on our institutional protocol, which follows standard practice for HDR-BT in locally advanced cervical cancer as supported by published guidelines. This initial dose level ensured adherence to widely accepted safety limits while allowing consistent applicator placement and reproducible treatment geometry. The chart of 7 Gy per session (Figure 1) illustrates the average D90 of each patient’s HR-CTV for the BT component.

According to the descriptive statistics (Figure 1), the group yielded a mean value of 6.70 ± 0.62 Gy (95% CI: 6.31-7.09 Gy). The SD of 0.618 and a variance of 0.382 reflect the treatment variability within this initial protocol.

For patient number 10, the mean D90 exceeds the prescribed dose, indicating improved tumor targeting. However, overdose may increase toxicity risk to adjacent OARs, requiring data to confirm safety. For all other patients, mean D90 falls below the target threshold, though generally remains aligned with therapeutic limits.

**Figure 2:**
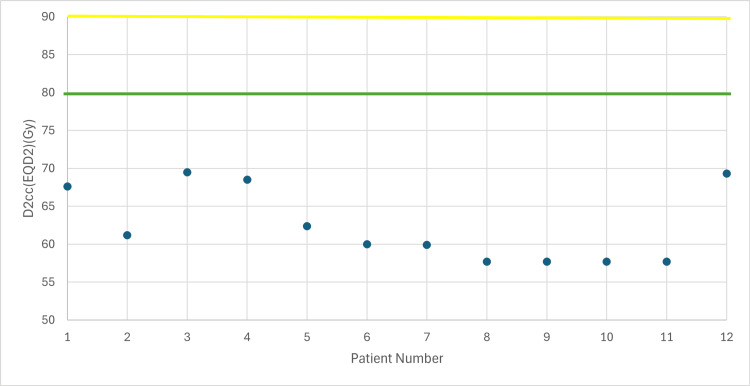
Total dose in bladder taking into account EBRT (EQD2) with 7 Gy prescribed dose D2cc: dose received by the 2 cc volume of each organ at risk, EBRT: external beam radiotherapy, EQD2: equivalent dose Yellow line: acceptable upper limit for the bladder according to the EMBRACE II protocol Green line: ideal upper limit for the bladder according to the EMBRACE II protocol

**Figure 3:**
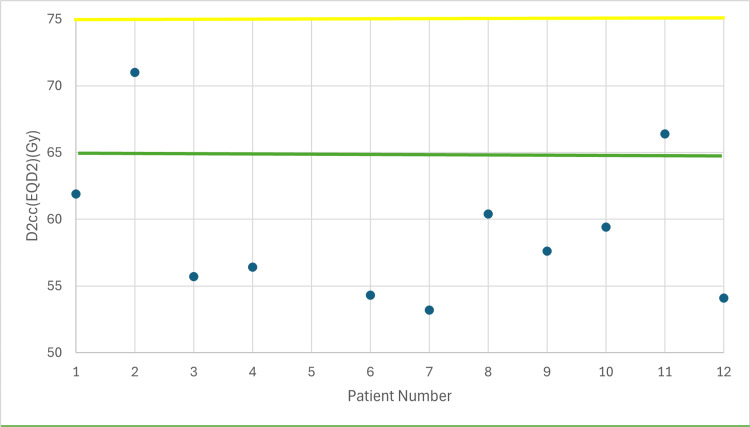
Total dose in rectum taking into account EBRT (EQD2) with 7 Gy prescribed dose D2cc: dose received by the 2 cc volume of each organ at risk, EBRT: external beam radiotherapy, EQD2: equivalent dose Yellow line: acceptable upper limit for the rectum according to the EMBRACE II protocol Green line: ideal upper limit for the rectum according to the EMBRACE II protocol

**Figure 4:**
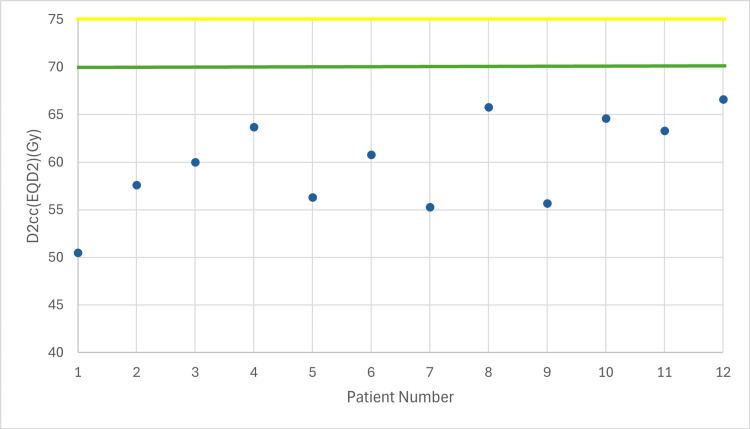
Total dose in sigmoid taking into account EBRT (EQD2) with 7 Gy prescribed dose D2cc: dose received by the 2 cc volume of each organ at risk, EBRT: external beam radiotherapy, EQD2: equivalent dose Yellow line: acceptable upper limit for the sigmoid according to the EMBRACE II protocol Green line: ideal upper limit for the sigmoid according to the EMBRACE II protocol

The following figures illustrate the total D2cc dose (EBRT+BT) for each OAR and evaluate how effectively the exposure to OARs is minimized according to the EMBRACE II protocol. The EQD2 equation was used to enable summation of doses from the two treatment plans (EBRT + BT). In Figures 2-4, the D2cc of each OAR is presented relative to two horizontal reference lines, the yellow line denoting the acceptable upper limit according to the EMBRACE II protocol, while the green line denotes the ideal upper limit. The acceptable upper limit is not exceeded for any patient, and instances where the ideal upper limit is surpassed are infrequent.

**Figure 5:**
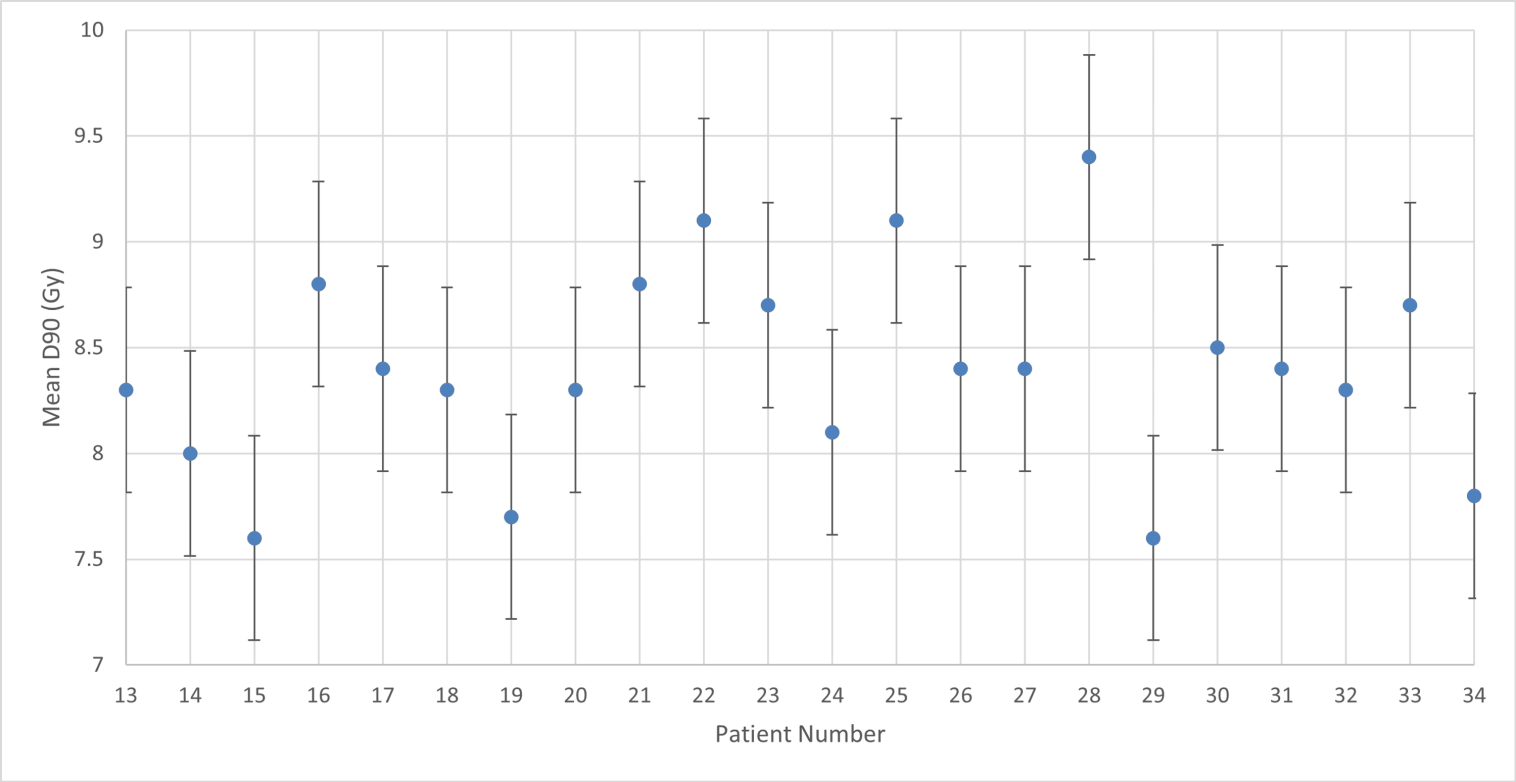
Mean D90 with 8 Gy prescribed dose D90: dose received by 90% of the high-risk clinical target volume

Data analysis was subsequently conducted on patients who received three 8 Gy BT sessions. While the 7 Gy protocol provided a safe baseline for OAR toxicity, it did not achieve optimal HR-CTV coverage, prompting the dose escalation. The chart of 8 Gy per session (Figure 5) illustrates the average D90 of each patient’s HR-CTV for the BT component.

According to the descriptive statistics (Figure 5), the group of 8 Gy per BT session yielded a significantly higher mean value of 8.40 ± 0.48 Gy (95% CI: 8.18-8.61 Gy). An independent samples t-test confirmed that this increase was highly significant compared to the 7 Gy group (t(32)=-8.855, p-value<0.001). Furthermore, a notable reduction in treatment variability was observed in the 8 Gy group, as evidenced by the lower SD (0.48 Gy vs. 0.62 Gy) and a narrower range (1.80 Gy vs. 2.40 Gy). Cohen's d effect size was calculated at 3.18, indicating a very large magnitude of effect for the dose escalation protocol. Normality of the data distribution was confirmed via the Shapiro-Wilk test (p>0.05).

It is apparent that, in most patients, the mean D90 value exceeds the prescribed dose, thereby confirming adequate coverage of the HR-CTV for the purpose of tumor eradication. However, in a few cases, the designed HR-CTV received a dose slightly below the prescription, primarily due to anatomical limitations and applicator positioning constraints that limited further dwell-time modulation during planning.

**Figure 6:**
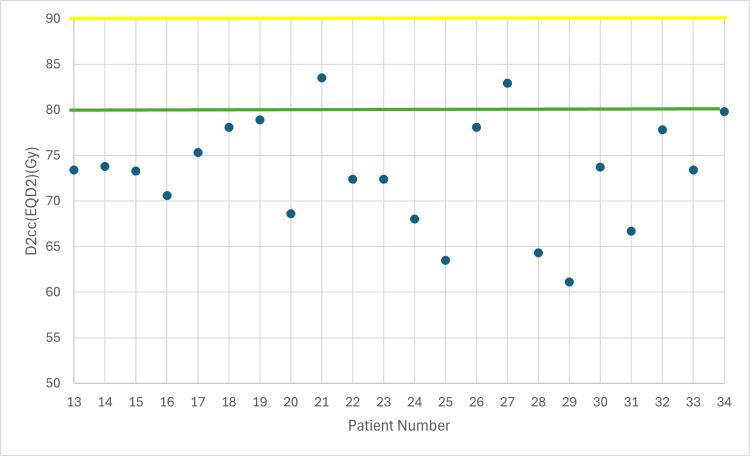
Total dose in bladder taking into account EBRT (EQD2) with 8 Gy prescribed dose D2cc: dose received by the 2 cc volume of each organ at risk, EBRT: external beam radiotherapy, EQD2: equivalent dose Yellow line: acceptable upper limit for the bladder according to the EMBRACE II protocol Green line: ideal upper limit for the bladder according to the EMBRACE II protocol

**Figure 7:**
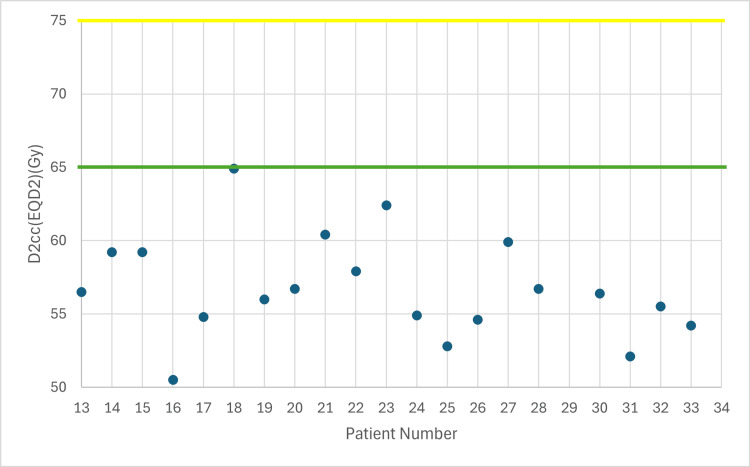
Total dose in rectum taking into account EBRT (EQD2) with 8 Gy prescribed dose D2cc: dose received by the 2 cc volume of each organ at risk, EBRT: external beam radiotherapy, EQD2: equivalent dose Yellow line: acceptable upper limit for the rectum according to the EMBRACE II protocol Green line: ideal upper limit for the rectum according to the EMBRACE II protocol

**Figure 8:**
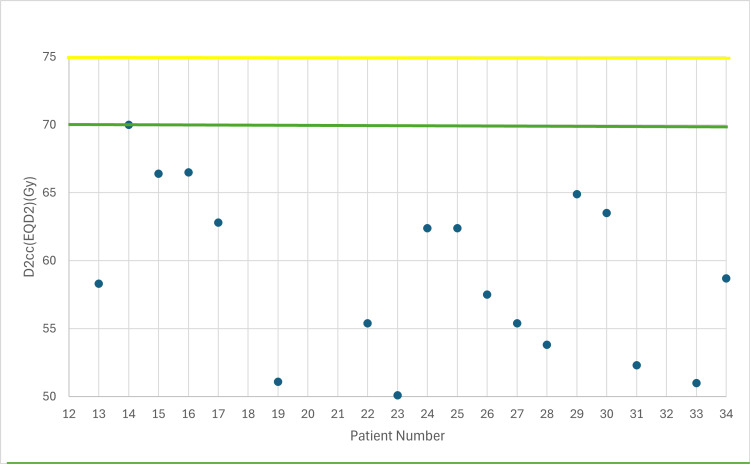
Total dose in sigmoid taking into account EBRT (EQD2) with 8 Gy prescribed dose D2cc: dose received by the 2 cc volume of each organ at risk, EBRT: external beam radiotherapy, EQD2: equivalent dose Yellow line: acceptable upper limit for the sigmoid according to the EMBRACE II protocol Green line: ideal upper limit for the sigmoid according to the EMBRACE II protocol

The following figures illustrate the total D2cc dose (EBRT+BT) for each OAR and evaluate how effectively the exposure to OARs is minimized according to the EMBRACE II protocol. The EQD2 equation was used to enable summation of doses from the two treatment plans (EBRT + BT). In Figures 6-8, the D2cc of each OAR is presented relative to two horizontal reference lines, the yellow line denoting the acceptable upper limit according to the EMBRACE II protocol, while the green line denotes the ideal upper limit. 

Despite increasing the prescribed dose from 7 to 8 Gy, the acceptable upper limit was not exceeded for any patient treatment, and instances where the ideal upper limit was surpassed remained infrequent, confirming the safety of the escalated protocol.

Total dose assessment

**Figure 9:**
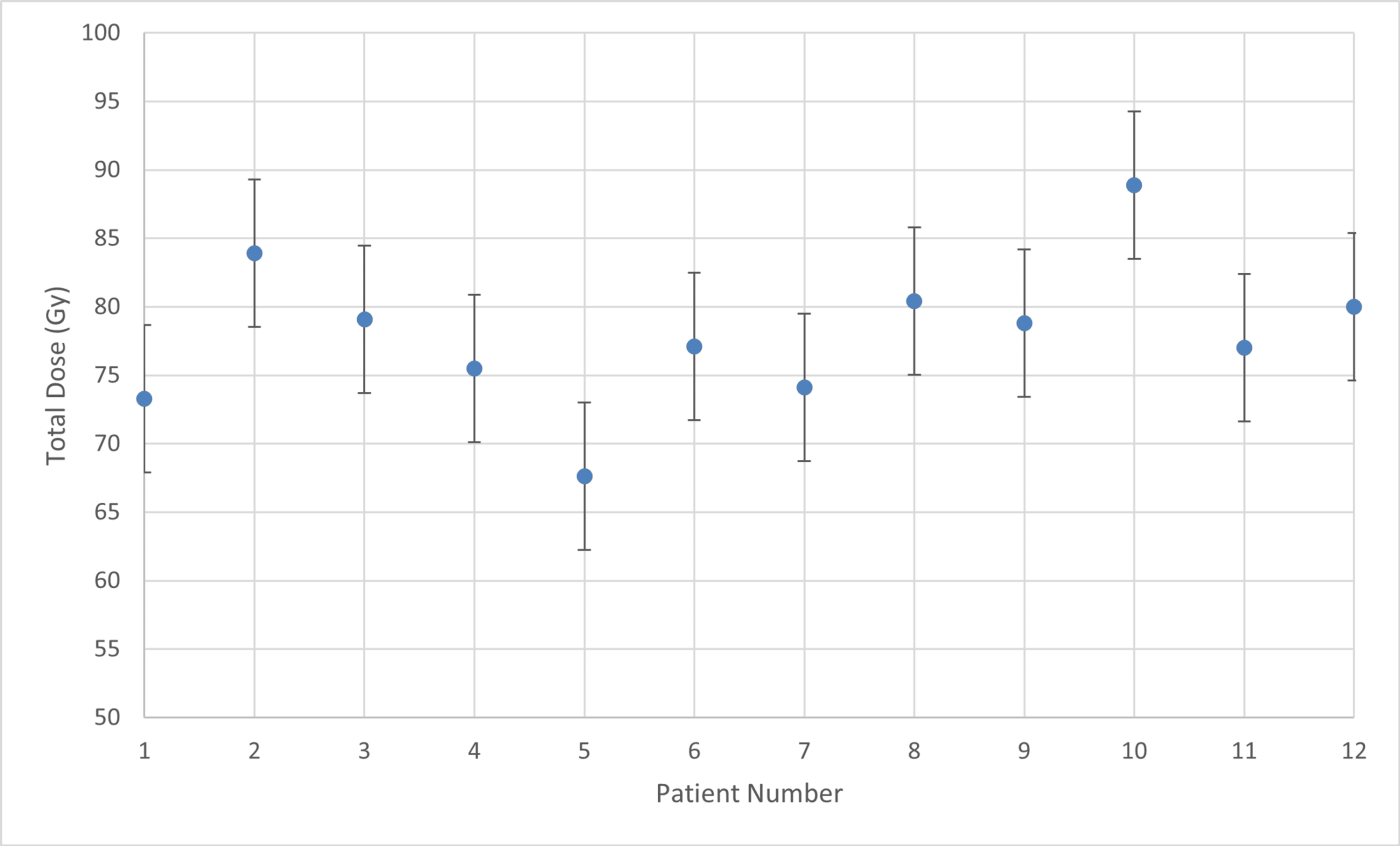
Total dose with 7 Gy prescribed dose per BT session BT: brachytherapy

In the next two figures, the aggregate cumulative dose delivered to the HR-CTV for each of the 34 patients is presented. This total dose was calculated by summing the EQD2 (α/β=10 Gy) contribution from the EBRT component and the three subsequent BT sessions. In Figure 9, the cumulative doses for the first group of 12 patients (treated with three BT sessions) are illustrated. 

**Figure 10:**
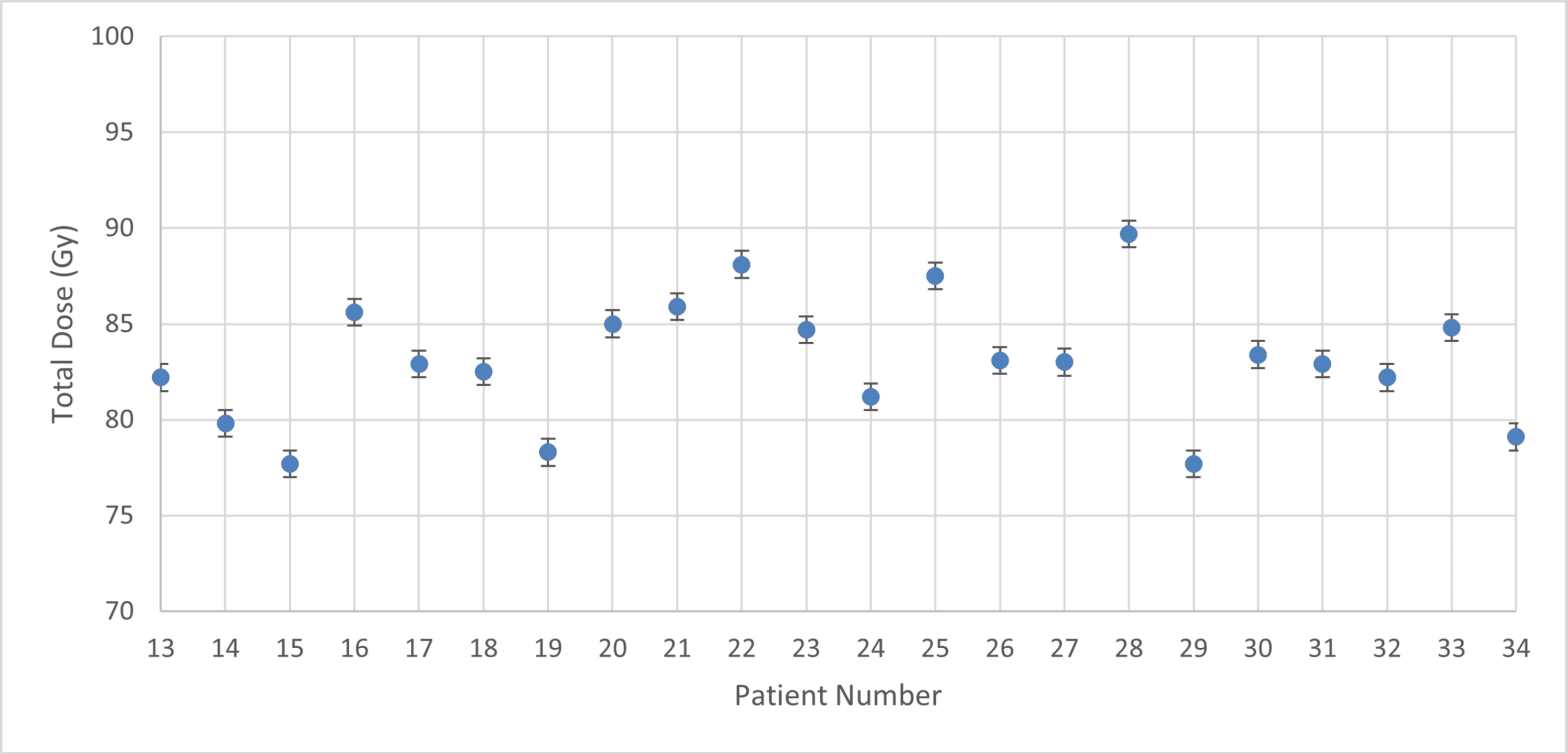
Total dose with 8 Gy prescribed dose per BT session BT: brachytherapy

On the contrary, Figure 10 presents the cumulative doses for the remaining 22 patients who underwent the dose-escalated protocol of three 8 Gy BT sessions.

The cumulative doses for the first group (Figure 9) of 12 patients (7 Gy per BT protocol) yielded a mean total dose of 77.98 ± 5.38 Gy (95% CI: 74.93-81.02 Gy). In contrast (Figure 10), the cumulative doses for the 22 patients in the 8 Gy group reached a significantly higher mean total dose of 83.06 ± 3.27 Gy (95% CI: 81.61-84.51 Gy). Notably, the SD decreased from 5.38 to 1.56, statistically confirming the reduction in treatment variability and the improved consistency of the dose-escalated protocol.

Regarding the overall cohort, seven patients achieved a total dose within the optimal 85-90 Gy interval, while thirteen patients fell within the 80-85 Gy range. Fourteen patients received a total dose below 80 Gy. Notably, eight out of twelve patients (66.7%) in the 7 Gy group failed to reach the 80 Gy threshold. In the 8 Gy group, only six patients remained below 80 Gy; these instances were primarily due to specific BT planning challenges, such as anatomical peculiarities and applicator positioning constraints that limited further dose optimization.

These results underscore that the transition to the 8 Gy protocol was instrumental in shifting the cumulative dose distribution toward the EMBRACE II recommended targets, significantly improving target coverage compared to the initial 7 Gy regimen.

## Discussion

Dose escalation

The escalation from 7 Gy to 8 Gy per BT session was not arbitrary, but it was guided by both the dosimetric findings of this study and established HDR-BT principles. With 7 Gy fractions, the D90 of the HR-CTV for several patients was close to the target limit of 7 Gy. Concurrently, the D2cc dose for each OAR remained within safe toxicity levels established by the EMBRACE II protocol. Increasing the dose to 8 Gy represented the smallest clinically meaningful step capable of achieving a consistent rise in D90 while maintaining all OAR D2cc values within EMBRACE II tolerance limits. This is aligned with standard HDR-BT practice, where dose adaptation is typically implemented in 1Gy increments due to their substantial EQD2 effect and favourable toxicity profile. In addition, the observed reduction in D90 variability following dose escalation (SD: 0.48 Gy vs. 0.62 Gy) can be attributed to improved dose conformity at higher prescription levels. At 8 Gy, the isodose distribution better encompassed the HR-CTV, reducing the sensitivity of D90 to anatomical variation and applicator-related uncertainties. This resulted in more homogeneous target coverage across patients and, consequently, narrower confidence intervals for the recorded D90 values (8.18-8.61 Gy for the 8 Gy Group vs. 6.31-7.09 Gy for the 7 Gy group). Collectively, the study highlights the critical need for dose escalation in cases where patients have large tumors or insufficient coverage, suggesting that such adjustments can enhance tumor control without compromising patient safety [14,15]. Nevertheless, an increased interstitial component, along with appropriate training and protocol adjustments, is essential to achieve a balance between tumor coverage and OAR sparing [16].

Total dose assessment

Upon evaluating the cumulative dose received by HR-CTV (EBRT+BT) for all 34 patients, the analysis of the data provided help to assess the feasibility of increasing the dose per BT session from 7 to 8 Gy, with the main interest being to approach 85 Gy cumulative dose in HR-CTV, while keeping doses in OARs below toxicity levels. A critical finding in this assessment was the marked reduction in treatment variability observed in the 8 Gy group. The SD for the total dose decreased from (5.38 Gy for the 7 Gy group) to (3.27 Gy for the 8 Gy group), statistically confirming that the dose-escalated protocol resulted in a more consistent and reproducible dose distribution across the patient cohort. In the initial 7 Gy group, the larger variability contributed to a higher rate of suboptimal coverage, with eight out of 12 patients (66.7%) failing to reach the 80 Gy threshold. In contrast, the 8 Gy protocol shifted the cumulative dose distribution toward the optimal 85-90 Gy interval, which was achieved by seven patients. Although a subset of patients still received a total dose below 85 Gy, this was attributable to specific limiting factors. Beyond the insufficient coverage inherent in the 7 Gy prescription, challenges in BT planning often stemmed from anatomical variations, difficulties in applicator placement, or previous pelvic surgical procedures. Furthermore, even with the 8 Gy protocol, three sessions occasionally proved insufficient for patients with extensive disease, particularly in stage IIIB cases. In such instances, the integration of interstitial (IS) BT with IU BT is required, involving the insertion of needles into the parametrial neoplastic tissue to achieve the necessary dose shaping and coverage.

Future direction

The comprehensive analysis conducted has yielded highly promising results, specifically concerning optimal tumor coverage and the safety of OARs, when passing from 7 Gy to 8 Gy per BT session treatment protocol. These results demonstrated that such an escalation is feasible within the strict OAR constraints defined by the EMBRACE II protocol. However, it must be emphasized that further increases in dose-per-fraction (e.g., to 9 Gy) or a reduction in the number of fractions are not recommended based on the current data. Such alterations would deviate from the EMBRACE II standardization and could significantly increase the risk of late toxicities, which cannot be assessed in this study due to the lack of long-term clinical follow-up [5]. Instead, future optimizations should focus on enhancing treatment precision through the transition from CT-based to MRI-based 3D BT planning. MRI guidance offers superior soft-tissue contrast for more accurate target delineation and is the current gold standard for identifying patients who may benefit from combined IC/IS BT techniques. Transitioning to MRI-based planning could represent a subsequent advancement to enhance the quality of BT and could determine whether further dose optimisation is clinically beneficial [17,18].

Limitations

The current analysis is constrained by several limitations. Its retrospective design and single-institution setting, along with a relatively small cohort size, may limit the external validity and generalizability of the findings. Furthermore, treatment planning was conducted solely using CT imaging, which offers inferior soft-tissue contrast compared to MRI-based guidance as recommended by EMBRACE II, thereby introducing potential uncertainties in target delineation and OAR contouring. In addition, the use of only three IC BT fractions per patient limits dosimetric optimization, especially in cases of large tumors where a combined IC/IS approach would be more appropriate [19]. Anatomical variability, challenges in applicator placement, and previous surgical procedures may also have confounded the delivered dose distributions. Lastly, the absence of longitudinal clinical outcomes, such as local control, late toxicity, and survival endpoints, prevents direct correlation of the dosimetric parameters with therapeutic efficacy and patient prognosis. More prospective studies with extended follow-up are necessary to validate the clinical safety of the 8 Gy escalation protocol [20,21].

## Conclusions

The primary objective of this study was to evaluate the feasibility and dosimetric impact of increasing the HDR-BT dose per session from 7 Gy to 8 Gy in cervical cancer treatment. The retrospective analysis of dosimetric data has yielded promising results regarding both tumor coverage and the safety of OARs, particularly following the dose escalation in BT sessions from 7 Gy to 8 Gy. This modification has enhanced tumor coverage without a significant increase in toxicity. Given the observed dosimetric benefits, future work may explore the feasibility of escalating the BT dose to 9 Gy per fraction or modifying the fractionation schedule. Such an investigation would require careful evaluation of OAR tolerance, tissue repair dynamics between fractions, and adherence to established clinical standards before any adjustment to clinical practice is considered. Additionally, transitioning from CT to MRI-based planning is anticipated as the subsequent step in optimizing treatment quality and efficacy.
